# Single-Cell, High-Content Microscopy Analysis of BK Polyomavirus Infection

**DOI:** 10.1128/spectrum.00873-23

**Published:** 2023-05-08

**Authors:** Megan C. Procario, Jonathan Z. Sexton, Benjamin S. Halligan, Michael J. Imperiale

**Affiliations:** a Department of Microbiology and Immunology, Medical School, University of Michigan, Ann Arbor, Michigan, USA; b Department of Internal Medicine, Medical School, University of Michigan, Ann Arbor, Michigan, USA; c Department of Medicinal Chemistry, College of Pharmacy, University of Michigan, Ann Arbor, Michigan, USA; d Center for Drug Repurposing, University of Michigan, Ann Arbor, Michigan, USA; e Rogel Cancer Center, University of Michigan, Ann Arbor, Michigan, USA; University of Wisconsin—Madison

**Keywords:** BKPyV, high-content microscopy, polyomavirus, single-cell infection

## Abstract

By adulthood, the majority of the population is persistently infected with BK polyomavirus (BKPyV). Only a subset of the population, generally transplant recipients on immunosuppressive drugs, will experience disease from BKPyV, but those who do have few treatment options and, frequently, poor outcomes, because to date there are no effective antivirals to treat or approved vaccines to prevent BKPyV. Most studies of BKPyV have been performed on bulk populations of cells, and the dynamics of infection at single-cell resolution have not been explored. As a result, much of our knowledge is based upon the assumption that all cells within a greater population are behaving the same way with respect to infection. The present study examines BKPyV infection on a single-cell level using high-content microscopy to measure and analyze the viral protein large T antigen (TAg), promyelocytic leukemia protein (PML), DNA, and nuclear morphological features. We observed significant heterogeneity among infected cells, within and across time points. We found that the levels of TAg within individual cells did not necessarily increase with time and that cells with the same TAg levels varied in other ways. Overall, high-content, single-cell microscopy is a novel approach to studying BKPyV that enables experimental insight into the heterogenous nature of the infection.

**IMPORTANCE** BK polyomavirus (BKPyV) is a human pathogen that infects nearly everyone by adulthood and persists throughout a person’s life. Only people with significant immune suppression develop disease from the virus, however. Until recently the only practical means of studying many viral infections was to infect a group of cells in the laboratory and measure the outcomes in that group. However, interpreting these bulk population experiments requires the assumption that infection influences all cells within a group similarly. This assumption has not held for multiple viruses tested so far. Our study establishes a novel single-cell microscopy assay for BKPyV infection. Using this assay, we discovered differences among individual infected cells that have not been apparent in bulk population studies. The knowledge gained in this study and the potential for future use demonstrate the power of this assay as a tool for understanding the biology of BKPyV.

## INTRODUCTION

BK polyomavirus (BKPyV) is a small, double-stranded-DNA tumor virus. It is one of approximately 14 human polyomaviruses (HPyVs). BKPyV infection, while usually asymptomatic, is very common and persists throughout a person’s lifetime. More than 90% of individuals are seropositive for BKPyV by the time they reach adulthood (1, 2). Despite its ubiquity, BKPyV’s disease burden is limited to patients posttransplant (reviewed in reference 3). Any person with significant immune defects may suffer from BKPyV-associated disease, but the most common are patients undergoing bone marrow, hematopoietic stem cell, or solid organ transplants. One complication that can occur in kidney transplant patients is polyomavirus-associated nephropathy (PVAN) due to new or reactivated BKPyV infection (4). The same immunosuppressant medications used to prevent rejection of the donor organs can prevent a sufficient immune response to clear the virus. There are currently no effective antivirals or approved vaccines to treat or prevent BKPyV (5–8). In these circumstances, clinicians must decide between decreasing the immunosuppression (and risk losing the graft) and maintaining immunosuppression (and risk uncontrolled viremia and destruction of the graft) (9, 10).

The BKPyV genome is circular, and transcription is driven by bidirectional promoters within a noncoding control region (NCCR), which also contains the origin of DNA replication, as well as transcription factor binding sites. One promoter drives early gene expression, which includes large T antigen (TAg), small t antigen, truncated tumor antigen, and Super T antigen (11–13). The other drives late gene expression of agnoprotein and capsid proteins VP1, VP2, and VP3. Upon entering the nucleus, the virus begins transcription of the early region, followed by translation and nuclear translocation of the proteins to facilitate viral DNA replication. The virus does so by stimulating the cell’s entry into S phase, recruiting host DNA replication machinery to the viral genome, and preventing apoptosis. BKPyV relies on manipulation of cell cycle regulation to facilitate efficient replication and both induces and relies upon the DNA damage response (DDR) for productive infection (14, 15). In conjunction with the onset of DNA replication, late genes are transcribed from the opposite promoter and the capsid proteins are translocated to the nucleus, where they self-assemble and package the nascent viral DNA to create progeny virions (13).

As demonstrated by its tumor antigens, BKPyV influences the behavior of many nuclear factors during the course of infection. These include promyelocytic leukemia nuclear bodies (PML-NBs) (16). PML-NBs are dynamic, punctate, subnuclear structures found in all mammalian cells. They are multiprotein complexes containing a scaffold of PML protein, the other constitutive component proteins, Sp100 and Daxx, and a number of other transiently associated proteins as circumstances require (17–18). These nuclear bodies are highly sumoylated and interact closely with cellular chromatin (19–21). PML-NBs respond to many forms of stress in the cell, ranging from metabolic deficiencies to senescence. Of particular relevance to BKPyV are their roles in both tumor suppression and antiviral pathways. Additionally, PML-NBs interact with the DDR pathway (22–26). BKPyV infection, along with that of many other viruses, has been demonstrated to significantly alter the structure and function of PML-NBs (reviewed in references 27 to 30).

The current *in vitro* model of primary renal proximal tubule epithelial (RPTE) cell infection has allowed many discoveries about BKPyV, including the viral life cycle, genetic regulation, and virus-host interactions (including PML-NBs) (16, 31–42). Almost all of these studies have been performed at the cell population level, and the results have generally been interpreted as being representative of any given individual cell in that population. However, a recent single-cell transcriptome sequencing (RNA-seq) study from the Pipas laboratory (43) has indicated that BKPyV infection is heterogeneous, with several discrete cellular states. In the present study, we sought to similarly examine single-cell phenotypes across the course of BKPyV infection using high-content microscopy. This morphological examination focused on three parameters: large T antigen (TAg), to identify BKPyV-positive cells and characterize the amount and distribution of this viral protein; PML, to identify structural changes in NBs; and DNA, as a marker of the nucleus, as well as to inform about the impact of infection on DNA and the nucleus in general. The high resolution and quantity of images allowed an advanced microscopic analysis of single-cell BKPyV infection that has not previously been attempted. These data provide insights into the heterogeneity of individual cells during the course of infection and offer suggestions for future mechanistic studies of BKPyV.

## RESULTS

The vast majority of our understanding of the biology of BKPyV has come from bulk-cell-population-level studies, many using the *in vitro* model of renal proximal tubule epithelial (RPTE) cells. Time course experiments have demonstrated these infections to follow a linear progression from cells expressing low levels of viral proteins at early time points to much greater amounts at later time points (13). However, a recent study examining BKPyV infection on a single-cell transcriptomic level has identified distinct subpopulations of infected cells (43). Cells displayed widely varying levels of viral transcripts within the population, and these differences were maintained as the infection progressed. Viral gene expression in cells did not always increase from low to high over time, further highlighting distinct subpopulations. Our study complements and expands on this newly emerging understanding of BKPyV infection at the single-cell level by focusing on morphological measurements using high-content confocal microscopy.

For our single-cell analysis of BKPyV infection, we chose to focus solely on nuclear features because BKPyV replicates and assembles in the nucleus. During the course of infection, nuclear host proteins, including PML, are modified and/or regulated. We sought to expand on our previous work that identified BKPyV infection as a cause of both structural and functional changes in PML-NBs (16). Specifically, we aimed to characterize the morphotype of PML-NB on a single-cell level throughout the course of BKPyV infection, paying particular attention to any correlation with TAg expression.

### Assay overview and imaging examples.

**FIG 1:**
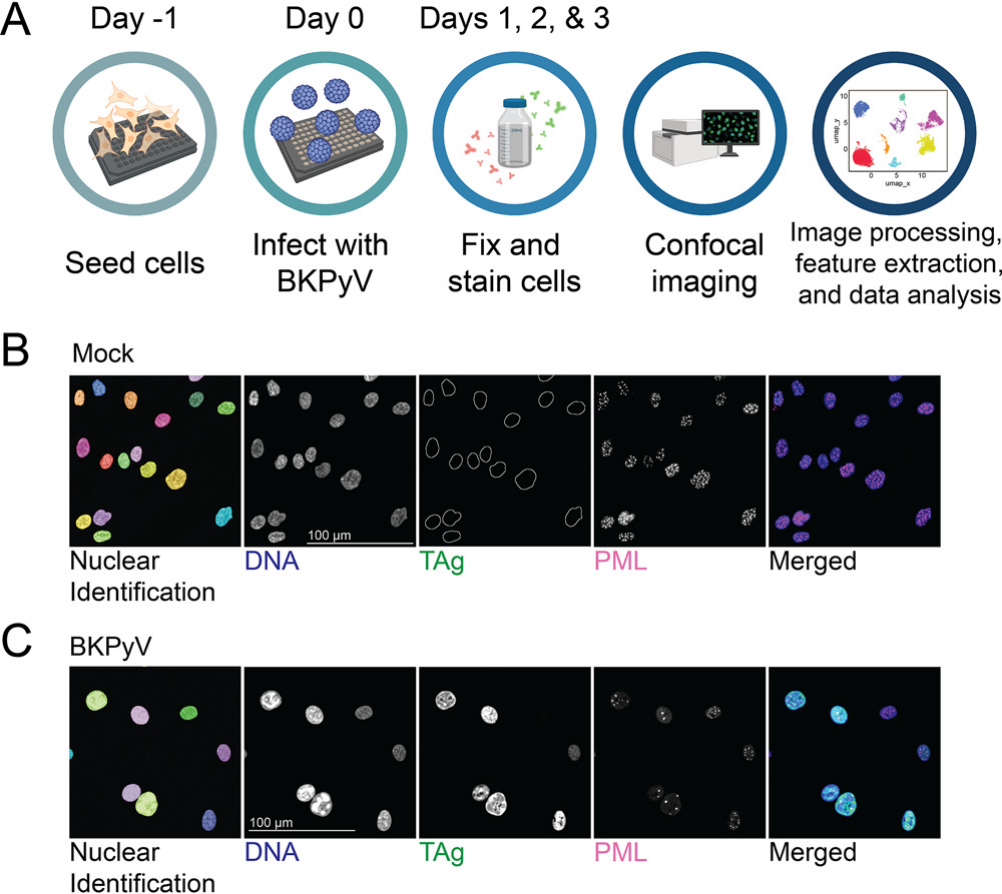
Assay overview and imaging examples. (A) Timeline illustrating the experimental protocol, the details of which can be found in Materials and Methods. (B and C) Representative images of cells at 2 days postinfection. The first panel indicates the nuclei as identified using the CellPose software, pseudocolored arbitrarily to distinguish individual objects. The next three panels are the individual channels, while the final panel is pseudocolored (DNA, blue; TAg, green; PML, magenta) and merged. (B) Cells from a mock-infected well. Nuclei are outlined in the TAg panel due to lack of visible signal. (C) Cells from a BKPyV-infected well.

To collect the image data, cells were infected at a multiplicity of infection (MOI) of 0.5 focus-forming units (FFU)/cell or mock infected and then fixed and stained after 1, 2, or 3 days (Fig. 1A). The early viral protein TAg and the principal component of PML-NBs, PML protein, were detected by immunofluorescence, and DNA was stained using Hoechst 33342 stain (Fig. 1B and C). We measured features relating to the objects’ size, shape, and area, as well as the magnitude, variation, and spatial distribution of signal intensity within each of the three channels (Tables S1 to S3 in the supplemental material). PML-NBs in each nucleus were identified, and select measurements were made for these as well.

### Single-cell microscopy as a method for studying BKPyV infection.

**FIG 2:**
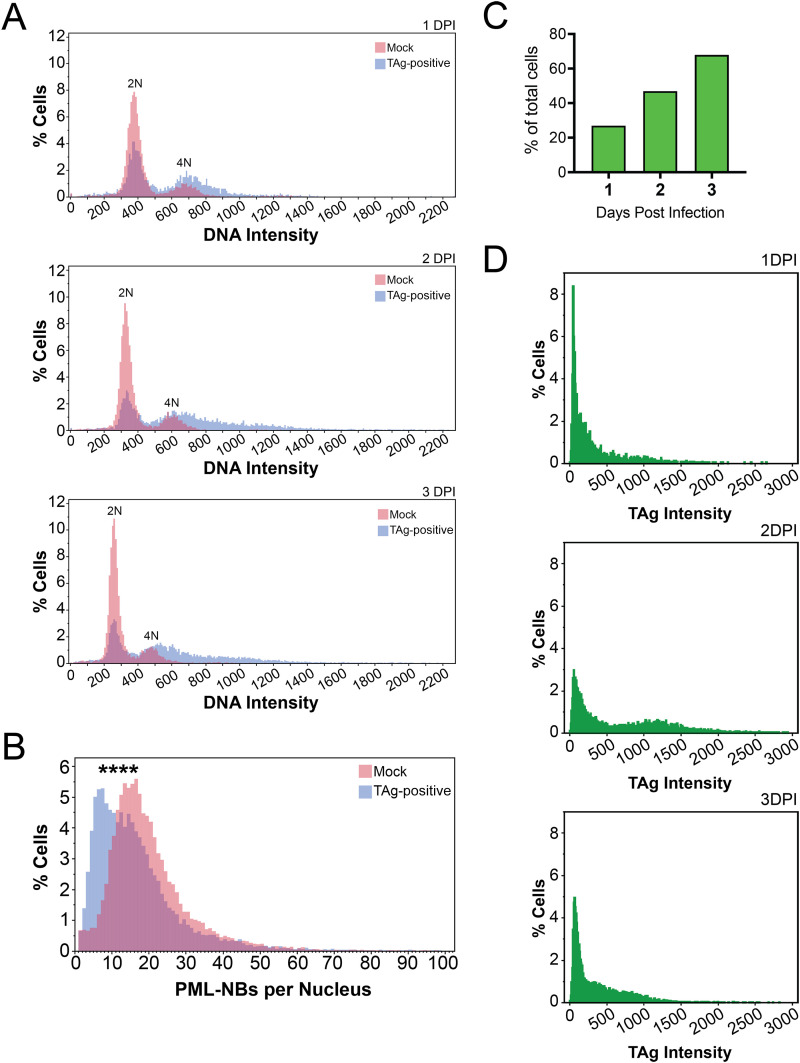
Single-cell microscopy as a method for studying BKPyV infection. (A) Histograms depicting the DNA contents of mock-infected (pink) and TAg-positive (blue) cells at the three time points. (B) As described for panel A but depicting the quantities of PML-NBs per nucleus in mock-infected (pink) and TAg-positive (blue) cells. (C) Percentages of TAg-positive cells out of total populations of cells at the indicated time points. (D) Histograms of only TAg-positive cells, depicting the distributions of cells with different levels of TAg at 1, 2, and 3 days postinfection. Wilcoxon one-way analysis: ***, *P* < 0.0001.

To validate the single-cell microscopy assay, we first examined a few well-established characteristics of BKPyV infection. Infection induces host cells to enter into S phase of the cell cycle and causes G_2_/M arrest, which increases the DNA content of infected cells (14, 43). BKPyV-infected cells have 4N DNA content or greater once they have progressed beyond S phase (44). To determine if our model demonstrated such an effect, we plotted histograms of the total DNA pixel intensity measurements from either the mock-infected cells or TAg-positive (TAg^+^) cells at each time point and examined the distribution of cells within each population. For mock-infected cells, the percentage of cells with >2N DNA content was approximately 21% for all 3 days. However, in the TAg^+^ cell population, this fraction of cells increased over time, to 30%, 45%, and 56% at 1, 2, and 3 days postinfection (dpi), respectively (Fig. 2A).

As an additional validation point, we quantified the number of PML-NBs per nucleus. BKPyV infection induces PML-NB rearrangement, which includes a reduction in the number of bodies (16). We plotted histograms of the distributions of numbers of PML-NBs per nucleus in the mock-infected and TAg^+^ cells at all time points (Fig. 2B). We found that 10% of mock-infected cells fell into the smallest bin of the histogram, with ≤8 nuclear bodies per nucleus, compared to 29% of TAg^+^ cells. This finding confirms our previous work but at a larger scale, as here we have analyzed roughly 40,000 cells, orders of magnitude greater than was feasible without high-content analysis (16).

Having validated that we were able to capture these two known host cell properties that change during infection, we next sought to focus on the characteristics of infected, TAg^+^ cells. First, we quantified the percentage of TAg^+^ cells at each time point and found that, as expected, this value increased with time (Fig. 2C). Next, we quantified the TAg signal intensities for each day postinfection (Fig. 2D). Consistent with the report from the Pipas laboratory, we found a wide range of TAg expression at all time points (43). While the data from days 1 and 3 are similar, with the majority of cells falling into low-TAg-intensity bins, the plot from day 2 had a bimodal distribution and a wider range. Unexpectedly, a greater percentage of cells at 2 dpi had overall higher TAg expression than those at 1 or 3 dpi.

### High DNA content, not time postinfection, distinguishes cells expressing the highest levels of TAg.

**FIG 3:**
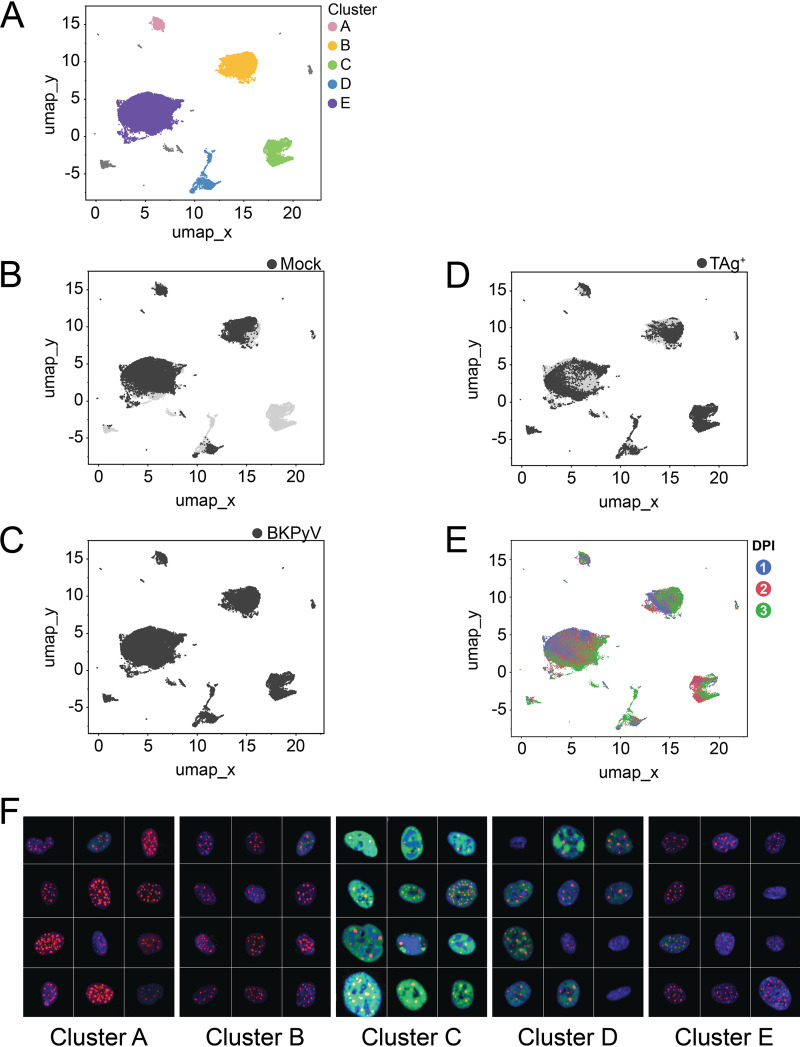
High DNA content, not time postinfection, distinguishes cells expressing the highest levels of TAg. (A) UMAP clusters from the global population were assigned a color and labeled with letters. (B) Mock-infected cells are highlighted in dark gray. (C) BKPyV-infected cells are highlighted in dark gray. (D) TAg-positive cells are highlighted in dark gray. (E) Cells are colored according to day postinfection. Blue, 1 dpi; red, 2 dpi; green, 3 dpi. (F) Montages of representative nuclei from the five global clusters of cells. Channels and images were normalized to allow comparison. Channel colors: TAg, green; PML, red; DNA, blue. Tile size is 150 pixels (px), or ~24.5 μm.

**FIG 4:**
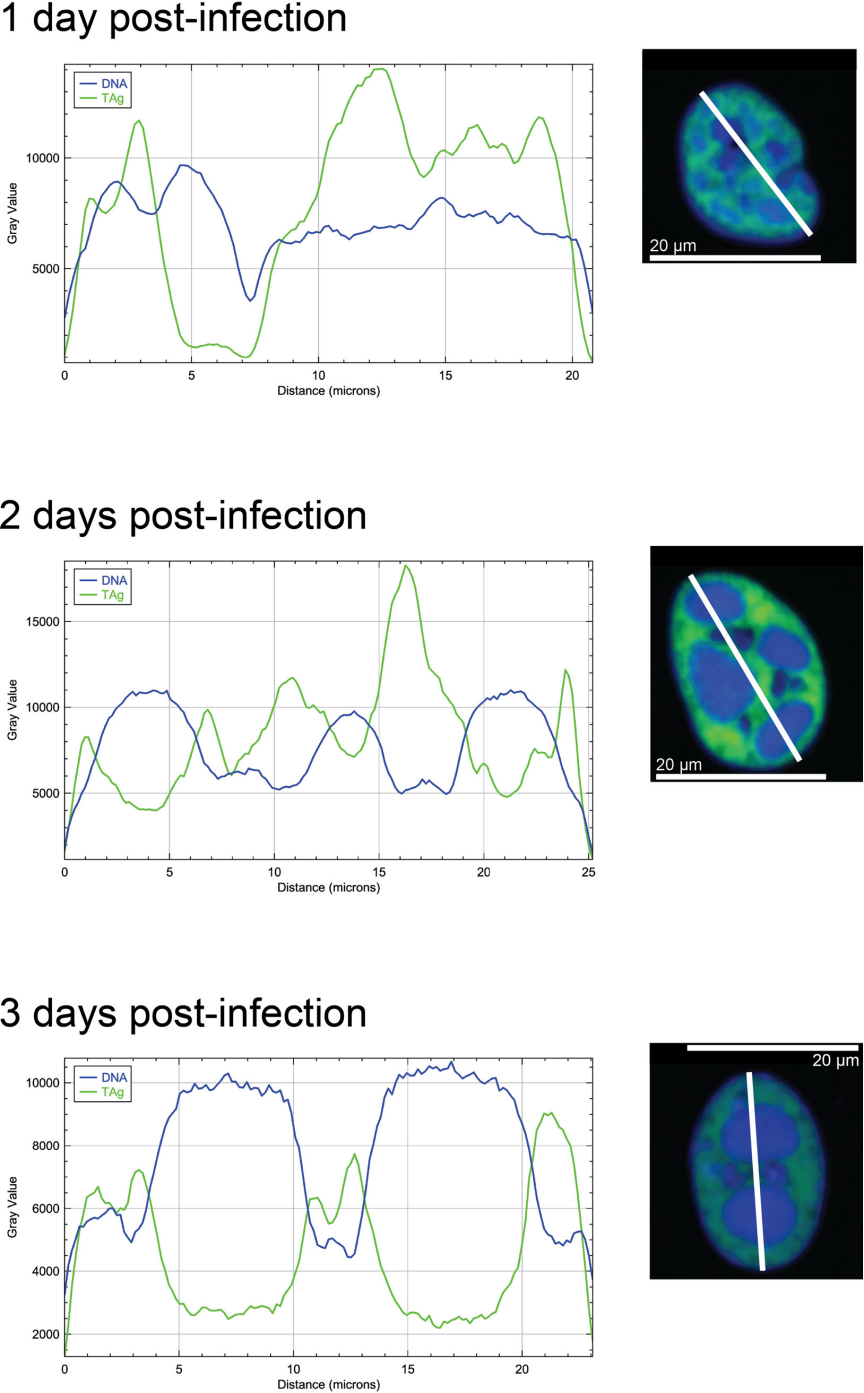
Appearance of cells with areas of mutual DNA/TAg exclusion. A representative nucleus was selected from each time point, and ImageJ software was used to plot the DNA and TAg intensities. Merged images of the two channels are shown. DNA, blue; TAg, green.

We next wanted to characterize the heterogeneity in infected cells. To visualize distinct morphologic cell classes, we employed dimensionality reduction and clustering using Uniform Manifold Approximation and Projection (UMAP) embedding. We started with a global UMAP, including all the cells regardless of infection or TAg status, and labeled the major clusters of cells alphabetically (Fig. 3A). It was immediately apparent that multiple distinct subgroups existed within the population of cells. Therefore, we explored the phenotypic features of all cells and differentiating factors in the subset of infected cells. First, to determine whether exposure to a viral inoculum defined the clusters, we highlighted the cells that received a mock inoculum (Fig. 3B) and BKPyV (Fig. 3C). Apart from cluster C, which was comprised only of cells that had received a viral inoculum, there did not appear to be any impact of infection condition on which cluster a cell was in. Second, we highlighted just the TAg^+^ cells to see if that better explained the clustering (Fig. 3D). Not all cells that received the viral inoculum expressed detectable TAg, and TAg^+^ cells were found in all the clusters. Interestingly, cluster C did not just exclusively contain infected cells, but also, almost all the cells were TAg^+^. We next asked if time postinfection was dictating some of the clustering. We colored cells on the UMAP by their day postinfection (Fig. 3E and Fig. S1A). Cells from each time point were present in every cluster, including cluster C, which had more cells at 2 days postinfection than at 1 or 3 dpi (Table S4). To determine which features in addition to TAg made cluster C distinct from the others, we used predictive modeling to identify the main contributors. The most significant feature was the integrated DNA intensity, which is proportional to the amount of DNA in the nucleus (Fig. S1B). Although other clusters had TAg^+^ cells and a range of DNA intensities, cluster C was distinct in having the highest DNA intensity overall. We assembled image montages of random cells from each of the clusters (Fig. 3F). These montages revealed patterns shared among cells that an observer might not notice if simply scanning a slide by eye. For example, we identified a phenotype in some cells in cluster C in which there appeared to be areas of very intense DNA signals within the nucleus. These areas of DNA intensity appeared to exhibit a much lower TAg signal than the rest of the nucleus, and the inverse was true as well. To examine this in more detail, we plotted the TAg and DNA intensities along a line drawn across the longest axis of a representative cell from each time point (Fig. 4). We observed that in cells with these areas of exclusion, the regions of high DNA intensity were anticorrelated with regions of high TAg intensity. This was especially noticeable at 2 and 3 dpi. PML-NBs were rarely identified within the intense DNA signal, but rather were found to be decorating the perimeter of these areas.

### TAg^+^ cells form clusters defined by features other than time point and TAg level.

**FIG 5:**
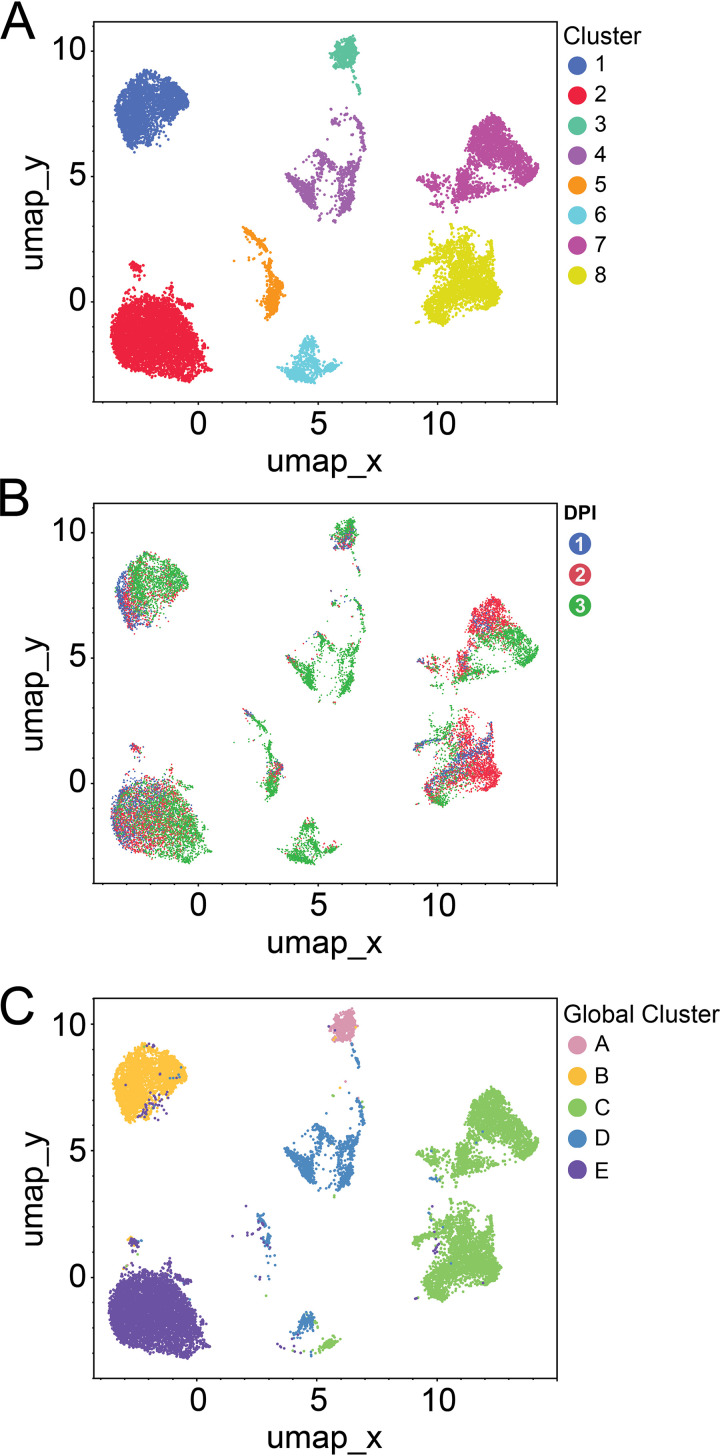
TAg^+^ cells form clusters defined by features other than time point and TAg level. (A) Clusters were identified from TAg-positive cells and labeled with numbers and different colors than the global clusters. (B) Individual cells’ time points were overlaid onto the positive UMAP. Blue, 1 dpi; red, 2 dpi; green, 3 dpi. (C) Positive UMAP with cluster colors from global UMAP overlaid.

We next explored the characteristics of the TAg^+^ cells by subsetting to TAg^+^ cells, reembedding that population in a new UMAP, and labeling the individual clusters numerically (Fig. 5A). Image montages for each of these eight clusters are shown in Fig. S2A. As with the global UMAP embedding, we wanted to see if the time postinfection was influencing the structure of the clusters by coloring the individual cells to represent the days postinfection (Fig. 5B and Fig. S2B). We observed that most individual clusters of TAg^+^ cells were not defined by time point. Two exceptions were cluster 6, which was almost entirely made up of cells at 3 days postinfection, and cluster 4, with nearly as high a percentage of 3-dpi cells as cluster 6 (Table S5). Instead of entire clusters being comprised of a single time point or a random distribution of time points, there appeared to be distinct substructures within each cluster for each day. Table 1 lists the top image features that define each cluster.

**TABLE 1:** Major defining features of TAg^+^ clusters

Cluster	Defining features
1	Lower DNA intensity, lower TAg intensity
2	Lower DNA intensity, lower DNA granularity, lower TAg intensity, more variation in PML signal
3	Lower DNA intensity, higher DNA granularity, smaller nuclei
4	Fewer PML-NBs/nucleus, slightly higher TAg granularity, lower PML intensity, less variation in PML signal, lower DNA intensity
5	Slightly higher TAg intensity, more PML-NBs per nucleus
6	Higher DNA intensity, lower PML intensity
7	High TAg intensity, PML signal infrequently overlaps DNA or TAg signal, fewer PML-NBs per nucleus
8	High TAg intensity, lower TAg granularity, higher PML intensity, slightly more PML-NBs/nucleus

To determine whether the TAg^+^ cell clustering in the global UMAP was reflected in the TAg^+^ UMAP, we plotted the TAg^+^ cell data using the TAg^+^ UMAP coordinates but color coded them using a given cell’s cluster assignment from the global UMAP (Fig. 5C). The TAg-positive cells in cluster B on the global UMAP were also a single cluster on the positive-only UMAP, cluster 1. The same was true for the TAg-positive cells from global cluster E, which were found on the TAg-positive UMAP in cluster 2. Global cluster D was largely represented in positive cluster 4, but it was also dispersed through clusters 3, 5, and 6. Global cluster C, which contained only TAg^+^ cells, was present but had become subdivided into two new clusters, clusters 7 and 8.

### Unique subpopulations exist among cells within the same TAg expression levels.

**FIG 6:**
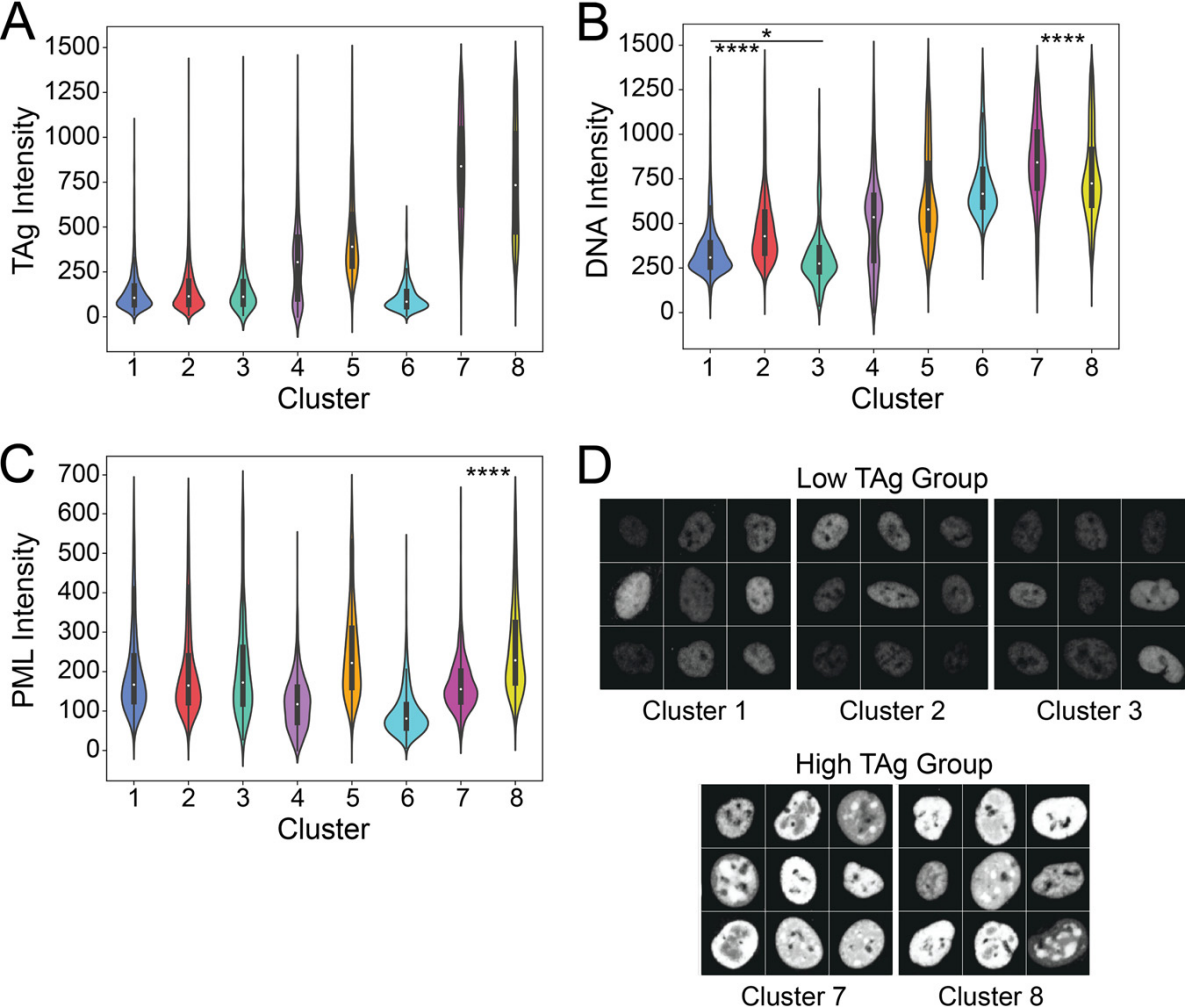
Unique subpopulations exist among cells within the same TAg expression levels. (A to C) Violin plots of per-nucleus levels of TAg (A), DNA (B), and PML (C) in TAg-positive cells. (D) Montages of randomly selected TAg-stained nuclei from the low-TAg (clusters 1 to 3) and high-TAg (clusters 7 and 8) groups of cells. Images were normalized to allow comparison across groups. Tile size is 150 px (~24.5 μm). Kruskal-Wallis multiple-comparison test: ****, *P* < 0.0001; *, *P* < 0.05.

To begin to understand what was defining the clusters in the TAg^+^-only UMAP, we generated violin plots of the TAg, DNA, and PML signal intensities for each cluster (Fig. 6A to C). We identified clusters that grouped together due to similar TAg intensities. Clusters 1, 2, and 3 all had low TAg levels, while clusters 7 and 8 had high TAg levels (Fig. 6A). Images of the TAg signal in randomly selected cells from the low-TAg group (clusters 1 to 3) and the high-TAg group (clusters 7 and 8) show the differences in TAg levels between the two groups (Fig. 6D). We next looked to see if clusters with the same TAg levels shared DNA intensity levels, but none of them did (Fig. 6B). Clusters 1, 2, and 3 were all significantly different from each other, with cluster 2 having the highest DNA content of that group. In the high-TAg group, we observed that cluster 7 had a greater DNA intensity than cluster 8. The PML intensity plot (Fig. 6C) showed that the low-TAg clusters, clusters 1 to 3, also shared PML expression levels. Clusters 7 and 8 were significantly different, however, with cluster 8 displaying the highest PML intensity of all the clusters.

**FIG 7:**
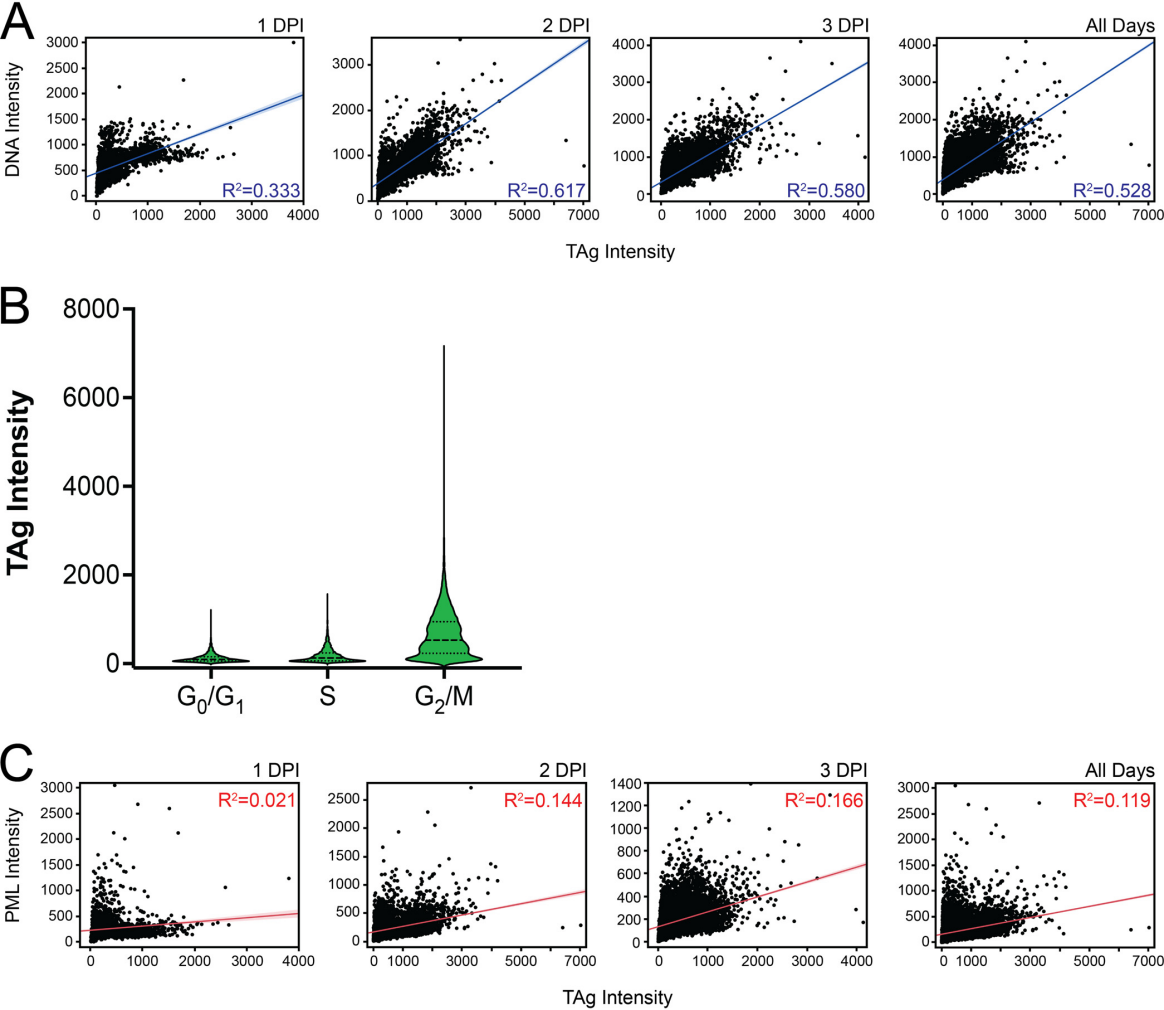
TAg correlation analyses. (A) Bivariate analysis to assess the correlation of DNA and TAg levels at each day postinfection and in the total population. *R*^2^ values are shown. (B) Distribution of TAg levels in cells in different phases of the cell cycle. (C) Bivariate analysis to assess the correlation of PML and TAg levels at each day postinfection and in the total population. *R*^2^ values are shown.

To better quantify the relationships between TAg expression and either DNA content or PML expression in the infected population, we performed bivariate analyses to determine the correlation between either macromolecule and TAg in a given cell. As we had observed different distributions of TAg expression at different time points, we performed this analysis on a per-time point basis and for the TAg^+^ population as a whole. We observed that there were statistically significant correlations (*P* < 0.0001) between DNA and TAg intensity levels at all times postinfection but that the strengths of these correlations were not the same at each time point: the correlation was greatest at 2 dpi, with an *R*^2^ value of 0.617 (Fig. 7A). In addition, the highest TAg levels were found in cells in G_2_/M phase (Fig. 7B). When we examined the relationship between PML and TAg levels, we found much smaller correlations (Fig. 7C), suggesting that very little of the variance seen in the PML signal was related to TAg.

### PML-NB characteristics account for differences between TAg expression groups.

**FIG 8:**
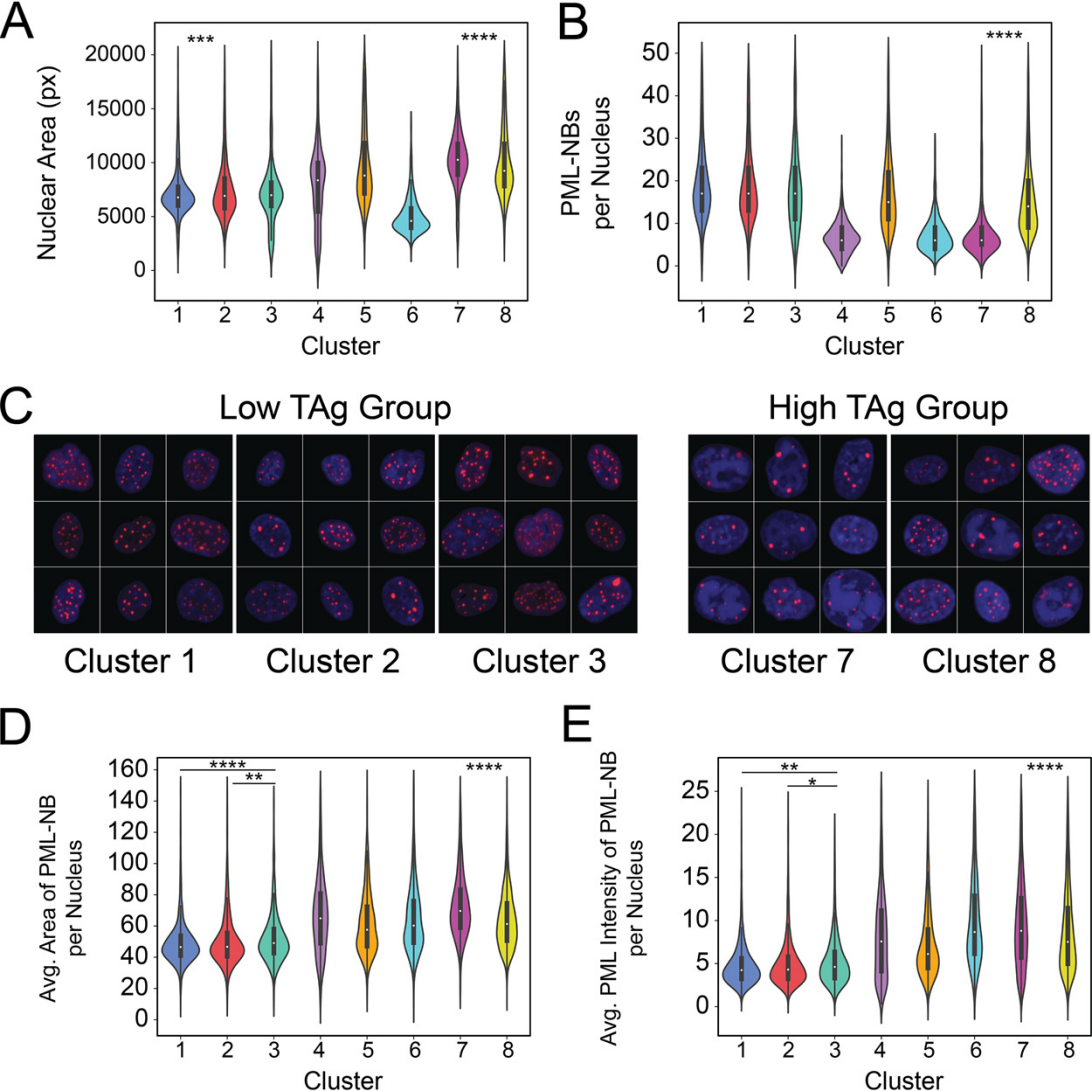
PML-NB characteristics account for differences between TAg expression groups. (A and B) Violin plots of per-nucleus values for nuclear area (A) and number of PML-NBs per nucleus (B) in TAg-positive cells. (C) Montages of randomly selected nuclei from the low-TAg (clusters 1 to 3) and high-TAg (clusters 7 and 8) groups of cells. Channels and images were normalized to allow comparison across groups. Channel colors: PML, red; DNA, blue. (D and E) Violin plots of PML-NB levels per nucleus for TAg-positive cells: average PML-NB area per nucleus (D); average PML intensity per NB (E). Tile size is 150 px (~24.5 μm). Kruskal-Wallis multiple-comparison test: ****, *P* < 0.0001; ***, *P* < 0.001; **, *P* < 0.01; *, *P* < 0.05.

To explore other differences that might be present between the low- and high-TAg groups, we quantified the nuclear area and the number of PML-NBs in each nucleus. In the low-TAg group, we found that the clusters had similarly sized nuclei, but clusters 1 and 2 were statistically different from each other, with cluster 2 having slightly larger nuclei (Fig. 8A). The nuclei in the high-TAg group were the largest, with cluster 7 having significantly larger nuclei than cluster 8 (Fig. 8A and Fig. S3A). We quantified the number of PML-NBs per nucleus and found that this number did not vary between clusters in the low-TAg group but that there was a difference in the numbers of PML-NBs per nucleus within the high-TAg group, with fewer PML-NBs per nucleus in cluster 7 than in cluster 8 (Fig. 8B and Fig. S3B). The difference in PML-NB numbers in the high-TAg group was particularly notable in images of the DNA and PML channels (Fig. 8C). While cluster 7 displayed a very homogeneous phenotype, cluster 8 was more of a mixture, mostly containing cells with many PML-NBs but with occasional low counts. This was readily apparent when graphed as a histogram (Fig. S3B). We also examined other features of the nuclear bodies within the individual clusters (Fig. 8D and E). We found that the low-TAg clusters had the lowest average areas of PML-NBs per nucleus and that cluster 3 had larger PML-NBs than clusters 1 and 2. In the high-TAg group, cluster 7 had larger PML-NBs than cluster 8 (Fig. 8D). In what is likely a correlate of PML-NB size, the intensity of the PML signal of the average PML-NB per nucleus was also lowest in the low-TAg group and was higher in cluster 7 than in cluster 8 (Fig. 8E).

## DISCUSSION

In establishing a single-cell, high-content microscopy assay of BKPyV infection, we have created a tool for studying a nonhomogeneous infection. The overall characteristics of infection were consistent with our understanding of BKPyV biology. We observed an increased population of cells with >2N DNA content and fewer PML-NBs per nucleus in the TAg^+^ cells than in the mock-infected cells. While the percentage of TAg^+^ cells increased over time, the TAg levels within individual cells did not increase in a linear fashion. We found that the distributions of TAg expression levels were much more similar at 1 and 3 days postinfection, with 2 days postinfection being the outlier with a greater range. Overall, while there was a notable correlation between DNA and TAg levels, TAg and PML protein levels appeared to have no impact on each other.

By quantifying the percentage of TAg^+^ cells at each time point in the experiment, we were able to see the contrast between looking at a bulk population and single cells. TAg positivity did follow the same pattern as the percentage of cells with >2N DNA content—increasing over time. The correlation between DNA and TAg levels was not as strong as bulk studies might suggest, but the time point with the maximum DNA-TAg correlation was also the time point with maximum TAg levels. Additionally, the highest TAg levels were present in cells in the G_2_/M phase of the cell cycle, as one might expect due to the DDR (14, 44). At 2 days postinfection, the range of distribution of TAg levels was greater than at other time points, and a greater percentage of 2-dpi cells had higher TAg levels. This was dramatically different enough to appear as a second peak that was not apparent at 1 or 3 days postinfection. This finding was in some contrast to the Pipas laboratory study, which identified high- and low-viral-protein-expressing populations of cells that were present by 2 dpi and remained until cell death (43). In our experiment, we would have expected to see the second peak on the histogram maintained through to the 3-day histogram (Fig. 2D). As the two laboratories used different MOIs and final endpoints, direct comparisons are difficult to make.

The machine learning approach together with UMAP dimensionality reduction and clustering that we used here has provided new insights into BKPyV infection. The presence or absence of TAg did not seem to influence the structure or identity of the clusters except for cluster C, for which TAg expression was the primary distinguishing factor. When focusing on the UMAP generated using only TAg^+^ cells, we observed that clusters could share TAg levels but had distinctions in DNA, PML, and PML-NB characteristics (Fig. 6). What was clear from the clustering, however, was that the response of cells to BKPyV infection varied from cell to cell.

The UMAP analyses revealed that in the global population, only the highest TAg levels defined a unique cluster of cells, cluster C. The other four clusters all contained some combination of TAg^+^, mock-infected, and TAg-negative cells. While time postinfection did not define most individual clusters, it did form distinct substructures within the clusters. Cells from a single day postinfection might not all cluster together, but within their given subpopulations, they were more similar to each other than they were to other cells in that cluster from different time points. This influence was not necessarily strong enough to overcome those features defining the clusters themselves, but it did shape the architecture within them.

Our prior work on BKPyV and PML-NBs demonstrated that infection caused a reorganization of PML-NBs, with an increase in the size and decrease in the number of NBs per nucleus (16). The prior study also established that, in a bulk population, BKPyV infection did not change the level of PML protein, suggesting that the TAg level did not correlate with the PML level. Here, we found that, despite having the same TAg levels, cells in the clusters had different PML protein levels and PML-NB arrangements, indicating that TAg levels and PML parameters were not directly related. This was supported by the very low correlation between TAg and PML levels at all times postinfection (Fig. 7C). While TAg itself might not account for the changes in PML-NBs, viruses Influence PML and PML-NBs through a myriad of mechanisms that could potentially be at play in our system. The simplest explanation is that PML was impacted directly by BKPyV genome replication, which is consistent with our previous findings (16). Another possible explanation is that BKPyV infection might be inducing changes in PML protein and/or PML-NBs indirectly as the NBs are interacting with the DDR in response to double-strand breaks and contributing to their repair. BKPyV infection induces and requires both arms of the DDR, mediated by ataxia telangiectasia mutated (ATM) and ATM and Rad3-related (ATR), for efficient replication and progeny production (14, 15). PML-NBs colocalize with Mre11 and Nbs1, members of the MRN (Mre11, Rad50, and Nbs1) complex, which is involved in sensing of double-stranded breaks and activation of the ATM pathway (45–47). Additionally, PML and PML-NBs support efficient homologous recombination (47, 48).

In the present study, within the group of high-TAg-expressing clusters, there were two distinct PML-NB phenotypes: cluster 7 displayed the previously described “reorganized” PML-NB arrangement (16), while cluster 8 had a more variable phenotype, with cells more likely to have more PML-NBs. It is possible that these two phenotypes represent distinct viral interactions with the DDR. Perhaps one phenotype is associated with sensing of DNA damage and recruitment of repair proteins, while the other is associated with active repair. Future studies are needed to test this possibility.

Many cells with high TAg levels displayed a TAg and DNA exclusion phenotype, wherein there were very distinct regions of intense DNA and TAg signals that occupied nonoverlapping regions of the nucleus. Cells with higher TAg and DNA contents were more likely to display this morphology. This intense DNA signal could represent densely packed heterochromatin, which has been reported to be regions of heterochromatin that are functioning as phase-separated domains (49, 50). These membrane-less structures within the nucleus have been demonstrated to regulate access to the DNA in ways beyond just volume exclusion. If the DNA is inaccessible due to phase separation, TAg may not be able to access it. This could mean that viral DNA replication, the initiation of which is mediated by TAg, is spatially separated from the host chromatin. The existence of this potential phase separation is supported by the observation that PML-NBs are generally found decorating the perimeter of these regions rather than within them, as other proteins may not occupy the same phase as the intensely stained DNA. Discrete TAg foci have previously been reported to be sites of viral DNA replication for other polyomaviruses (46, 51), but the function of the more diffuse-staining TAg is not known.

There is precedent for examining DNA and PML in a single-cell microscopic analysis of HPV infection, which showed great heterogeneity in infection kinetics among individual cells (52). Additionally, one of our laboratories has previously published results using a similar assay with severe acute respiratory syndrome coronavirus 2 (SARS-CoV-2) infection for a drug-repurposing screen (53), and other groups have published SARS-CoV-2 findings as well, including another drug-repurposing screen (54). The single-cell assay used in our study comes with both benefits and limitations. This approach facilitates the examination of BKPyV infection on a level that has not yet been widely explored. These tools have allowed us to increase the quantity of measurements and objects measured. Using machine learning has allowed us to take an unbiased approach to scoring our cells and performing other analyses. The high-resolution confocal imaging has facilitated the discovery of DNA-staining patterns in the nuclei of infected cells that had not yet been reported in the BKPyV literature. In the future, one will need to consider that observed increases in TAg levels in bulk populations may not represent equivalent increases in all the cells. At the transcriptional level, it has been demonstrated that only a small percentage of individual BKPyV-positive cells express high levels of viral genes (43). Individual cells could be identified as high- or low-viral-transcript producers by 2 days postinfection, and they did not progress beyond those states. In conjunction with our findings, the transcript data reinforce that there are likely gaps in our understanding of polyomavirus infection that can only be filled by studying single-cell infection.

There are limitations as well. For this initial study, we chose to examine only a small number of macromolecules. Therefore, we cannot draw direct conclusions about any factors beyond TAg, DNA, or PML. Additionally, while we have gained meaningful insights about differences between clusters of cells with high TAg levels, we have not yet been able to draw clear conclusions about some of the other clusters. We were able to identify that clusters 1 to 3 all had the same quite low TAg levels, but beyond that, their differences were subtle. However, when looking at what defined them as individual clusters, all three had DNA features whose biological meaning is currently unclear at the top of their lists. Cluster 6 was even more influenced by DNA features, with its top 4 of 5 defining characteristics being various measurements of DNA content. Cluster 4 had the greatest variety of defining characteristics, which may be reflected in its unique shape. Along with cluster 5, it was the most challenging to interpret. Examining other parameters beyond our current three will help clarify what defines and shapes these cells in the absence of an obvious interpretable defining feature like high TAg.

Our results are only an introduction to what can be done using high-content automated microscopy and machine learning analysis. In the future, we would like to expand both the size and scope of the assay to include more time points and more macromolecules, including a more in-depth examination of the interaction between PML-NBs and the DDR. One could additionally explore the areas of intense DNA staining, paying particular attention to markers of chromatin. The addition of the single-cell microscopy assay to the current arsenal of tools will facilitate a more thorough and clearer understanding of viral infections.

## MATERIALS AND METHODS

### Cell culture.

RPTE cells (catalog number CC-2553; Lonza) were grown in renal epithelial cell basal medium (REBM) (catalog number CC-3191; Lonza) supplemented with SingleQuots (catalog number CC-4127; Lonza) to make renal epithelial cell growth medium (REGM) and passaged up to six times as previously described (55). RPTE-hTERT cells (56) were also maintained in REBM/REGM.

293TT cells (57) were maintained in Dulbecco’s modified Eagle’s medium (DMEM) with 10% fetal bovine serum and 100 U/mL penicillin, 100 μg/mL streptomycin as described in Broekema et al. (58). All cells were grown in a humidified incubator at 37°C and 5% CO_2_.

### Virus growth and titration.

The BKPyV Dunlop variant was propagated as described previously (58), with some modifications. Briefly, the linearized Dunlop genome was digested from the pGEM backbone at flanking BamHI sites. Gel purification was performed, followed by circularization of the genome. The remainder of the protocol proceeded as described previously.

Viral titers were determined using a modified version of the previously described fluorescent focus assay (FFA) (55). Subconfluent RPTE-hTERT cells were seeded on tissue culture-treated glass chamber slides. The next day, the cells were infected with either a medium-only control or 10-fold serial dilutions of purified virus, with a 1-h adsorption at 4°C and medium replacement following that. Forty-eight hours postinfection, the cells were fixed with 4% paraformaldehyde and probed for TAg (PAb416, 1:500 dilution). After the cells were incubated with the secondary antibody, DNA was stained with 1 μg/mL Hoechst 33342 stain and coverslips were mounted using Prolong glass antifade mountant (catalog number P36982; Invitrogen). Images were acquired at ×20 magnification using an Olympus BX60 camera. ImageJ software (59) was used to quantify the number of TAg-positive cells per well. Cells were only considered positive if the TAg signal was brighter than any found in the mock control and if it was present in the nucleus. Viral titers were calculated as the mean values from three wells.

### Infection and immunofluorescence staining.

Using 96-well plates with optically clear bottoms (item number 655090; Greiner), 8,000 RPTE-hTERT cells per well were seeded, avoiding the perimeter wells. The outside wells were filled with phosphate-buffered saline (PBS) to prevent evaporation of medium from those containing cells. The day after seeding, the cells were incubated at 4°C for 15 min and then either mock infected or infected at an MOI of 0.5 in 40 μL per well of REGM. The cells were returned to 4°C for a 1-h adsorption. After the adsorption, the inoculum was aspirated from the wells and replaced with 100 μL of pre-warmed REGM per well. The cells were returned to 37°C for 1, 2, or 3 days. At the appropriate time point, medium was aspirated from the cells and replaced with room temperature 4% paraformaldehyde for a 20-minute incubation (40 μL per well). The wells were washed thoroughly with PBS after fixation, especially if the plate was being returned to the incubator for later time points. Following the fixation and wash, the cells were permeabilized and blocked for 4 h in 40 μL per well of blocking buffer at room temperature. The buffer was aspirated from the cells and replaced with 40 μL per well of primary antibodies in antibody dilution buffer. The cells were incubated in primary antibody overnight at 4°C. The following day, cells were washed three times in PBS and then incubated with secondary antibodies and 10 μg/mL Hoechst 33342 stain for 2 h at room temperature. After this final incubation step, the cells were washed three more times with PBS, and then the wells were filled with 100 μL PBS each for imaging. Details of all antibodies used in this study can be found in Table 2. For all immunofluorescence staining, blocking steps were performed using blocking buffer (1× PBS, 5% goat serum, 0.3% Triton X-100) and antibody dilution steps were performed in antibody dilution buffer (1× PBS, 1% BSA, 0.3% Triton X-100). All washes were done using 1× PBS without calcium or magnesium.

**TABLE 2:** Antibodies used in this study

Antibody	Host	Dilution range	Use^*a*^	Reference or source
Primary antibodies				
PAb416 (anti-TAg)	Mouse	1:250–1:1,000	IFA and FFA	65
PML Ab (PG-M3)	Mouse	1:250–1:500	IFA	sc-966; Santa Cruz
Secondary antibodies				
Anti-rabbit IgG–Alexa Fluor 594	Goat	1:250–1:1,000	IFA	A11012; Invitrogen
Anti-mouse IgG–Alexa Fluor 488	Goat	1:250–1:1,000	IFA	A11001; Invitrogen
Anti-mouse IgG–Alexa Fluor 594	Goat	1:250–1:1,000	FFA	A11005; Invitrogen

a IFA, immunofluorescence assay; FFA, fluorescent focus assay.

### High-content imaging.

All imaging was performed using a confocal Yokogawa Cell Voyager 8000 (CV8000) high-content imaging system with a water immersion UPSLAPO 40×/1.0 numeric aperture (NA) objective. The optical configuration and fluorophores for the time course experiment were as follows: for nuclei, Hoechst 33342 stain (405-nm laser, 445/45 emission filter); for TAg, Alexa Fluor 488 (488-nm laser, 525/50 emission filter); and for PML, Alexa Fluor 594 (561-nm laser, 600/37 emission filter). Laser power and exposure times were adjusted to optimize signal-to-background readings. Due to the high-NA objectives and thin depth of focus, maximum-projection images were collected over a 10-μm Z-range at 1-μm intervals to ensure that all cells were in focus. Thirty-two fields per well were collected to ensure an adequate number of cell observations. Postacquisition background and geometry correction was performed for each channel.

### Image identification, feature extraction, infection classification, and curve fitting.

Individual nuclei in the images of Hoechst 33342 staining were identified and nuclear masks were generated in open-source CellPose 2.0 software, using a mean cell diameter of 110, CellPose’s native edge removal to prevent generating masks of partial cells, and the built-in Cyto-2 pretrained model (60, 61). Nuclear masks were imported into CellProfiler 4.2.1 along with the fluorescence images for feature extraction (62). PML-NBs were identified within the nuclei using Otsu’s adaptive two-class thresholding with a size range of 3 to 20 pixels. Intensity features were tabulated for each fluorescence channel, along with size/shape measurements for nuclei and PML-NBs, resulting in 668 total features per nucleus.

All cells were classified as TAg positive or negative with CellProfiler Analyst 3.0.4 (63) using a semisupervised machine learning approach. Approximately 100 each of TAg-positive and -negative cells were hand selected, and a random forest (RF) classifier model was trained using 5-fold cross-validation. The classification model resulted in high accuracy (>99.6%) on the test set. The RF feature importance was tabulated, and as expected, the top features included those that measured TAg signal intensity.

For the signal intensity plots, single-channel grayscale images from either the DNA or TAg channel were opened using ImageJ software. A bisecting line was drawn down the longest axis of the selected cell at the same coordinates in each image. The PlotProfile function was run for both images, and a plot was generated.

### Data processing and UMAP embedding.

To systematically explore the morphologies of BKPyV-infected cells, UMAP dimensionality reduction was performed similarly to the procedure of Mirabelli et al. (53). The embed_UMAP application of MPLearn (version 0.1.0; https://github.com/maomlab/MPLearn) was used to generate UMAP embeddings. Briefly, for a set of cells, each feature was standardized using the scikit-learn StandardScaler. Then, features were reduced to two dimensions with the umap-learn python package originally described in McInnes et al., using the defaults listed in the documentation (64). Embeddings were visualized using JMP 16 software, and CellProfiler Analyst was used to generate single-nucleus tiled images of selected clusters. The subset of TAg^+^ cells was selected and reembedded to further characterize the morphologic and intensity characteristics of the infected cell population.

### Statistical testing.

Wilcoxon one-way and Kruskal-Wallis multiple-comparison analyses were performed using either JMP or Prism.
