# Supplementary material for: Single-Cell, High-Content Microscopy Analysis of BK Polyomavirus Infection
Source: Microbiol Spectr. 2023 May 8;11(3):e00873-23. doi: 10.1128/spectrum.00873-23 (PMC10269497; doi:10.1128/spectrum.00873-23)

Procario et al., Supplmental Materials

Supplemental Table 1
Nuclear measurements obtained by Cell Profiler software*

AreaShape	AreaShape	AreaShape	Correlation
Area	*MajorAxisLength*	*Zernike_0_0*	Correlation_DNA_PML
BoundingBoxArea	*MaxFeretDiameter*	*Zernike_1_1*	Correlation_DNA_TAg
BoundingBoxMaximum_X	*MaximumRadius*	*Zernike_2_0*	Correlation_PML_TAg
BoundingBoxMaximum_Y	*MeanRadius*	*Zernike_2_2*	Costes_DNA_PML
BoundingBoxMinimum_X	*MedianRadius*	*Zernike_3_1*	Costes_DNA_TAg
BoundingBoxMinimum_Y	*MinFeretDiameter*	*Zernike_3_3*	Costes_PML_DNA
CentralMoment_0_0	*MinorAxisLength*	*Zernike_4_0*	Costes_PML_TAg
CentralMoment_0_1	*NormalizedMoment_0_0*	*Zernike_4_2*	Costes_TAg_DNA
CentralMoment_0_2	*NormalizedMoment_0_1*	*Zernike_4_4*	Costes_TAg_PML
CentralMoment_0_3	*NormalizedMoment_0_2*	*Zernike_5_1*	K_DNA_PML
CentralMoment_1_0	*NormalizedMoment_0_3*	*Zernike_5_3*	K_DNA_TAg
CentralMoment_1_1	*NormalizedMoment_1_0*	*Zernike_5_5*	K_PML_DNA
CentralMoment_1_2	*NormalizedMoment_1_1*	*Zernike_6_0*	K_PML_TAg
CentralMoment_1_3	*NormalizedMoment_1_2*	*Zernike_6_2*	K_TAg_DNA
CentralMoment_2_0	*NormalizedMoment_1_3*	*Zernike_6_4*	K_TAg_PML
CentralMoment_2_1	*NormalizedMoment_2_0*	*Zernike_6_6*	Manders_DNA_PML
CentralMoment_2_2	*NormalizedMoment_2_1*	*Zernike_7_1*	Manders_DNA_TAg
CentralMoment_2_3	*NormalizedMoment_2_2*	*Zernike_7_3*	Manders_PML_DNA
Compactness	*NormalizedMoment_2_3*	*Zernike_7_5*	Manders_PML_TAg
ConvexArea	*NormalizedMoment_3_0*	*Zernike_7_7*	Manders_TAg_DNA
Eccentricity	*NormalizedMoment_3_1*	*Zernike_8_0*	Manders_TAg_PML
EquivalentDiameter	*NormalizedMoment_3_2*	*Zernike_8_2*	Overlap_DNA_PML
EulerNumber	*NormalizedMoment_3_3*	*Zernike_8_4*	Overlap_DNA_TAg
Extent	*Orientation*	*Zernike_8_6*	Overlap_PML_TAg
FormFactor	*Perimeter*	*Zernike_8_8*	RWC_DNA_PML
HuMoment_0	*Solidity*	*Zernike_9_1*	RWC_DNA_TAg
HuMoment_1	*SpatialMoment_0_0*	*Zernike_9_3*	RWC_PML_DNA
HuMoment_2	*SpatialMoment_0_1*	*Zernike_9_5*	RWC_PML_TAg
HuMoment_3	*SpatialMoment_0_2*	*Zernike_9_7*	RWC_TAg_DNA
HuMoment_4	*SpatialMoment_0_3*	*Zernike_9_9*	RWC_TAg_PML
HuMoment_5	*SpatialMoment_1_0*		
HuMoment_6	*SpatialMoment_1_1*		
InertiaTensorEigenvalues_0	*SpatialMoment_1_2*		
InertiaTensorEigenvalues_1	*SpatialMoment_1_3*		
InertiaTensor_0_0	*SpatialMoment_2_0*		
InertiaTensor_0_1	*SpatialMoment_2_1*		
InertiaTensor_1_0	*SpatialMoment_2_2*		
InertiaTensor_1_1	*SpatialMoment_2_3*		

*Each measurement was obtained once for each object. Those listed in italics were also obtained for each PML-NB.

Supplemental Table 2
Nuclear measurements obtained by Cell Profiler software[#]

Granularity	Intensity	RadialDistribution	Texture*
Granularity_1	*IntegratedIntensityEdge*	FracAtD_1of4	AngularSecondMoment
Granularity_2	*IntegratedIntensity*	FracAtD_2of4	Contrast
Granularity_3	*LowerQuartileIntensity*	FracAtD_3of4	Correlation
Granularity_4	*MADIntensity*	FracAtD_4of4	DifferenceEntropy
Granularity_5	*MassDisplacement*	MeanFrac_1of4	DifferenceVariance
Granularity_6	*MaxIntensityEdge*	MeanFrac_2of4	Entropy
Granularity_7	*MaxIntensity*	MeanFrac_3of4	InfoMeas1
Granularity_8	*MeanIntensityEdge*	MeanFrac_4of4	InfoMeas2
Granularity_9	*MeanIntensity*	RadialCV_1of4	InverseDifferenceMoment
Granularity_10	*MedianIntensity*	RadialCV_2of4	SumAverage
	MinIntensityEdge	RadialCV_3of4	SumEntropy
	MinIntensity	RadialCV_4of4	SumVariance
	StdIntensityEdge	ZernikeMagnitude_0_0	Variance
	StdIntensity	ZernikeMagnitude_1_1	
	UpperQuartileIntensity	ZernikeMagnitude_2_0	
		ZernikeMagnitude_2_2	
		ZernikeMagnitude_3_1	
		ZernikeMagnitude_3_3	
		ZernikeMagnitude_4_0	
		ZernikeMagnitude_4_2	
		ZernikeMagnitude_4_4	
		ZernikeMagnitude_5_1	
		ZernikeMagnitude_5_3	
		ZernikeMagnitude_5_5	
		ZernikeMagnitude_6_0	
		ZernikeMagnitude_6_2	
		ZernikeMagnitude_6_4	
		ZernikeMagnitude_6_6	

[#]Each measurement was obtained once for each object in each channel. Those listed in italics were also obtained for each PML-NB.

* *All textures measured at a scale of 4 and 12 pixels and in all four directions*

Supplemental Table 3
PML-NB measurements obtained by Cell Profiler software

Location	Neighbors	RadialDistribution
CenterMassIntensity_X	AngleBetweenNeighbors_Expanded	FracAtD_PML_1of3
CenterMassIntensity_Y	FirstClosestDistance_Expanded	FracAtD_PML_2of3
MaxIntensity_X	FirstClosestObjectNumber_Expanded	FracAtD_PML_3of3
MaxIntensity_Y	NumberOfNeighbors_Expanded	MeanFrac_PML_1of3
	PercentTouching_Expanded	MeanFrac_PML_2of3
	SecondClosestDistance_Expanded	MeanFrac_PML_3of3
	SecondClosestObjectNumber_Expanded	RadialCV_PML_1of3
		RadialCV_PML_2of3
		RadialCV_PML_3of3

Supplemental Table 4
Percentages of cells at the different time points in each global cluster

	**Cluster**				
	**A**	**B**	**C**	**D**	**E**
1 DPI	39%	32%	13%	23%	32%
2 DPI	20%	24%	48%	18%	36%
3 DPI	42%	44%	38%	59%	32%

Supplemental Table 5
Percentages of cells at the different time points in each TAg^+ cluster

	Cluster							
	**1**	**2**	**3**	**4**	**5**	**6**	**7**	**8**
1 DPI	19%	17%	25%	4%	14%	1%	8%	20%
2 DPI	21%	32%	17%	6%	22%	4%	41%	57%
3 DPI	61%	51%	59%	90%	65%	95%	51%	24%

Supplemental Figure Legends

Supplemental Figure 1. Cluster C contains more cells at 2 dpi. A. Global UMAP with cells colored by day post infection as in figure 3E. Each day has been highlighted separately to better demonstrate the distribution of timepoints within clusters. B. Violin plot of per-nucleus DNA content in each cluster.

Supplemental Figure 2. Distribution of TAg-positive cells in the clusters. A. Montages of representative nuclei from the eight TAg-positive clusters of cells. Channels and images are normalized to allow for comparison. Channel colors: TAg = green, PML = red, DNA = blue. Tile size is 150 px (~ 24.5 µm). B. TAg-positive UMAPs with cells colored by day post infection as in figure 5B. Each day has been highlighted separately to better demonstrate the distribution of timepoints within clusters.

Supplemental Figure 3. Clusters in the high TAg group differ in multiple ways including nuclear area and PML-NB quantity. A. Histogram depicting the area of the nuclei demonstrated in the violin plot in figure 7A, but only from Clusters 7 and 8. B Histogram depicting the quantity of PML-NBs per nucleus as was graphed via violin plot in figure 7B, only for Clusters 7 and 8. In both histograms Cluster 7 is represented in green, while Cluster 8 is represented in purple.

Supplemental Figure 1

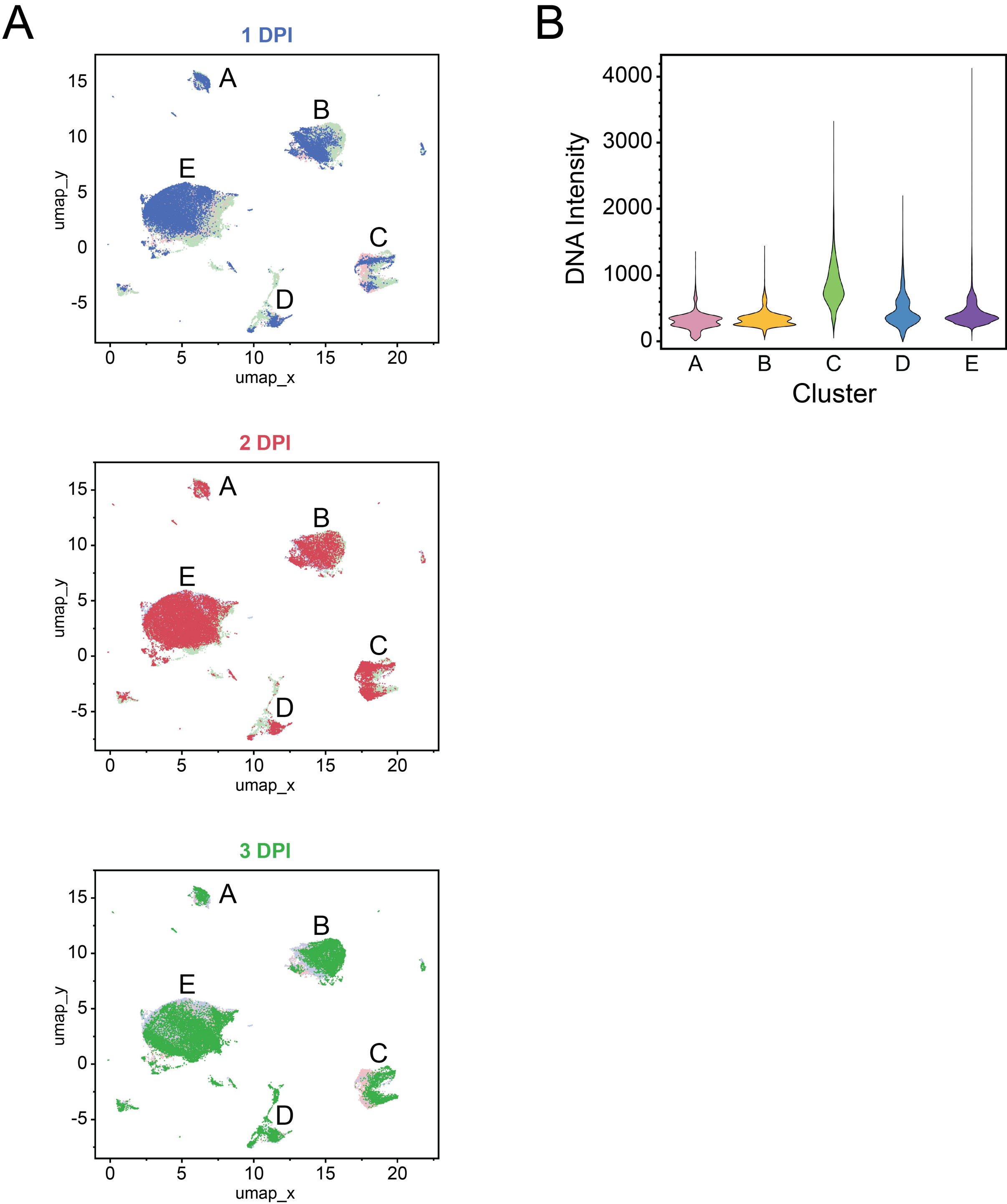

Supplemental Figure 2

A

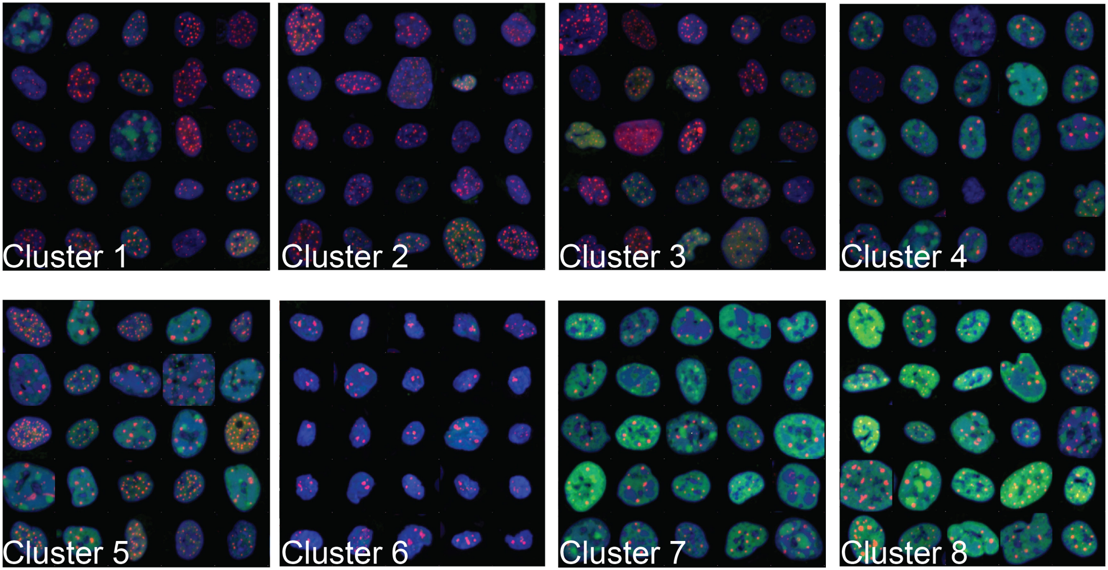

B

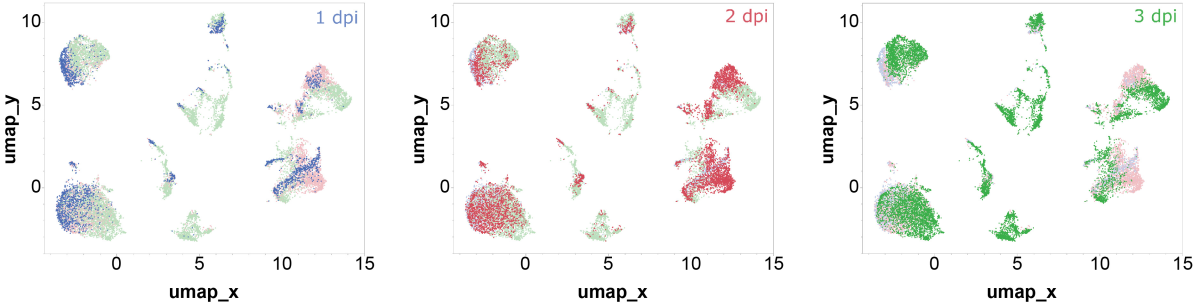

Supplemental Figure 3

A

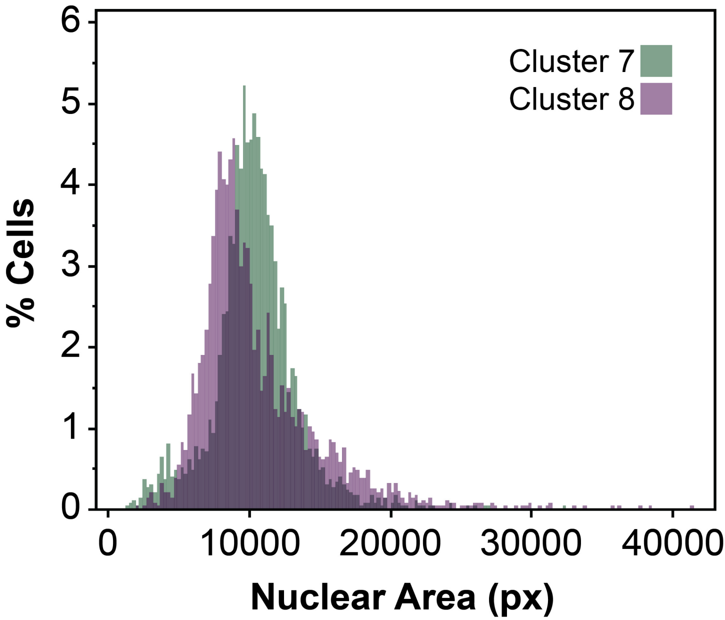

B

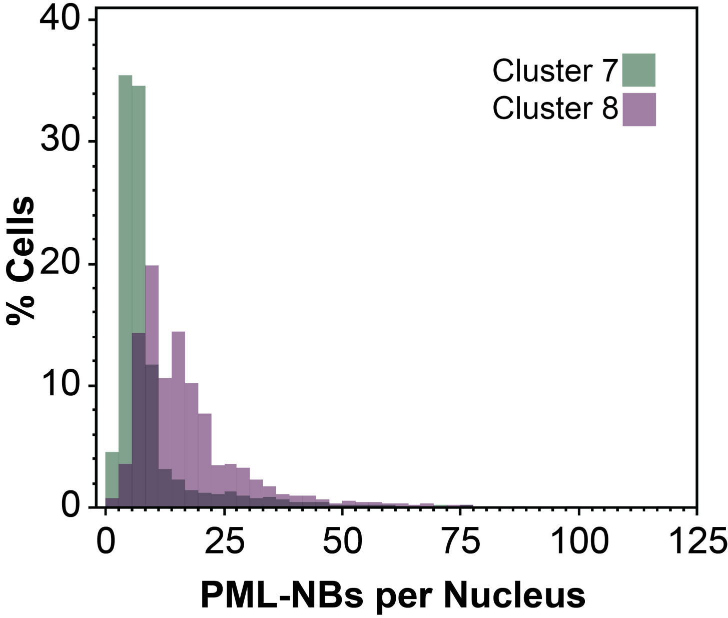

Supplement: Supplemental file 1 — Supplemental material. Download spectrum.00873-23-s0001.pdf, PDF file, 4.1 MB [file spectrum.00873-23-s0001.pdf]
